# Retraction: Silencing lncRNA XIST exhibits antiproliferative and proapoptotic effects on gastric cancer cells by up-regulating microRNA-132 and down-regulating PXN

**DOI:** 10.18632/aging.205702

**Published:** 2024-03-15

**Authors:** Ping Li, Liuhua Wang, Pengfei Li, Fangyong Hu, Yi Cao, Dong Tang, Gang Ye, Hongbo Li, Daorong Wang

**Affiliations:** 1 Department of General Surgery, Huaian Tumor Hospital, Huaian Hospital of Huaian City, Huaian 223200, P.R. China; 2 Department of Experimental Surgery-Cancer Metastasis, Medical Faculty Mannheim, Ruprecht Karls University, Mannheim 68167, Germany; 3 Department of General Surgery, Northern Jiangsu Province Hospital, Clinical Medical College, Institute of General Surgery - Yangzhou, Yangzhou University, Yangzhou 225000, P.R. China; 4 Department of General Surgery, Jiangdu People's Hospital of Yangzhou, Yangzhou 225200, P.R. China

**Keywords:** gastric cancer, long non-coding RNA XIST, microRNA-132, paxillin, proliferation

**This article has been retracted:** Aging has completed its investigation of this paper. We found internal duplication in **Figure 2C**, illustrating results of wound healing assays, and duplication between EdU assay images in **Figures 2B** and **3G**. We also found multiple instances of overlap between some of the EdU assay images used for **Figures 2B, 3G** and **5D** and data published by other authors in 2018 and 2019 [1–4]. Unexpectedly, the images of rulers (with specific imperfections) used to illustrate tumor size duplicate such images from two unrelated papers [5,6]. Consequently, all authors agreed that the article should be retracted.
